# Analysis of the Peptidoglycan Hydrolase Complement of *Lactobacillus casei* and Characterization of the Major γ-D-Glutamyl-L-Lysyl-Endopeptidase

**DOI:** 10.1371/journal.pone.0032301

**Published:** 2012-02-27

**Authors:** Krzysztof Regulski, Pascal Courtin, Mickael Meyrand, Ingmar J. J. Claes, Sarah Lebeer, Jos Vanderleyden, Pascal Hols, Alain Guillot, Marie-Pierre Chapot-Chartier

**Affiliations:** 1 Institut National de la Recherche Agronomique, UMR1319 Micalis, Jouy-en-Josas, France; 2 AgroParisTech, UMR Micalis, Jouy-en-Josas, France; 3 Centre of Microbial and Plant Genetics, K.U. Leuven, Leuven, Belgium; 4 Biochimie et Génétique Moléculaire Bactérienne, Institut des Sciences de la Vie, Université Catholique de Louvain, Louvain-la-Neuve, Belgium; University of Kansas Medical Center, United States of America

## Abstract

Peptidoglycan (PG) is the major component of Gram positive bacteria cell wall and is essential for bacterial integrity and shape. Bacteria synthesize PG hydrolases (PGHs) which are able to cleave bonds in their own PG and play major roles in PG remodelling required for bacterial growth and division. Our aim was to identify the main PGHs in *Lactobacillus casei* BL23, a lactic acid bacterium with probiotic properties.

The PGH complement was first identified *in silico* by amino acid sequence similarity searches of the BL23 genome sequence. Thirteen PGHs were detected with different predicted hydrolytic specificities. Transcription of the genes was confirmed by RT-PCR. A proteomic analysis combining the use of SDS-PAGE and LC-MS/MS revealed the main seven PGHs synthesized during growth of *L. casei* BL23. Among these PGHs, LCABL_02770 (renamed Lc-p75) was identified as the major one. This protein is the homolog of p75 (Msp1) major secreted protein of *Lactobacillus rhamnosus* GG, which was shown to promote survival and growth of intestinal epithelial cells. We identified its hydrolytic specificity on PG and showed that it is a γ-D-glutamyl-L-lysyl-endopeptidase. It has a marked specificity towards PG tetrapeptide chains versus tripeptide chains and for oligomers rather than monomers. Immunofluorescence experiments demonstrated that Lc-p75 localizes at cell septa in agreement with its role in daughter cell separation. It is also secreted under an active form as detected in zymogram. Comparison of the muropeptide profiles of wild type and Lc-p75-negative mutant revealed a decrease of the amount of disaccharide-dipeptide in the mutant PG in agreement with Lc-p75 activity. As a conclusion, Lc-p75 is the major *L. casei* BL23 PGH with endopeptidase specificity and a key role in daughter cell separation. Further studies will aim at investigating the role of Lc-p75 in the anti-inflammatory potential of *L. casei* BL23.

## Introduction

Peptidoglycan (PG) is the major cell wall component in Gram positive bacteria, and plays a key role to guarantee bacterial cell integrity and shape. PG is constituted of glycan chains made of alternating β-1,4-linked *N*-acetyl-glucosamine (GlcNAc) and *N*-acetyl-muramic acid (MurNAc) cross-linked by peptidic chains which composition varies between bacterial species [1]. The cell wall PG is the target of specific PG hydrolases (PGHs) (also named autolysins), synthesized by the bacteria themselves [2], [3]. According to their hydrolytic bond specificity, PGHs are classified into different classes: *N*-acetylmuramidases and *N*-acetylglucosaminidases which hydrolyze glycan chains between MurNAc and GlcNAc or GlcNAc and MurNAc, respectively, *N*-acetylmuramyl-L-Ala amidases which hydrolyze the bond between MurNAc and the first L-Ala of the lateral peptidic chain, and peptidases which hydrolyze different bonds inside PG peptidic chains. In addition, lytic transglycosylases break the same bonds as muramidases but yield anhydromuropeptides as products. D-Ala-carboxypeptidases are also usually present but are not able to provoke bacterial lysis on their own thus they are not classified as autolysins. These PGHs are involved in different cellular functions in bacteria, which require cell wall remodelling during growth and division. These include cell wall expansion, peptidoglycan turn over, recycling and maturation, and daughter cell separation. In conditions leading to growth arrest, their PG hydrolysing activity is susceptible to provoke bacterial lysis.

Lactobacilli are important components of the natural flora of the human digestive tract and certain strains are also recognized as probiotic bacteria. Probiotics are defined as live microorganisms which, when ingested in sufficient amount, have health benefits besides their traditional nutritional effects. The most obvious positive effects attributed to lactobacilli include their impact on immune responses, in particular the down-regulation of inflammatory responses through the induction of anti-inflammatory mechanisms mediated by immunocompetent cells and by their secreted cytokines [4], [5].

*Lactobacillus casei* BL23 strain has been reported to stimulate human peripheral blood mononuclear cells (PBMCs) to produce anti-inflammatory cytokine IL-10 [6]. In addition, it was shown to have anti-inflammatory properties *in vivo* in murine models of intestinal inflammation and to protect mice in TNBS- as well as in DSS-induced colitis [6], [7]. However, knowledge of the molecular and cellular mechanisms involved in these effects is still lacking. As reported previously in other lactobacilli strains [8], [9], PG metabolism and muropeptide release by PGHs could modulate immune cell response and cytokine production. Therefore we have undertaken to characterize the major PGHs in *L. casei* BL23.

In this study, we determined *in silico* the PGH complement of *L. casei* BL23 and identified by a proteomic analysis the main PGHs synthesized by bacteria during exponential growth phase. We characterized in detail the major PGH, LCABL_02770 (renamed Lc-p75), by biochemical and genetic studies.

## Results

### *In silico* search of the PGH complement of *L. casei* BL23

The PGH complement of *L. casei* BL23 was identified *in silico* in the available BL23 genome sequence [10] by amino acid sequence similarity search with well known PGHs from other Gram positive species [3]. Thirteen putative enzymes were identified with different predicted hydrolytic specificities (Table 1). Among these, two putative amidases LCABL_11280 and LCABL_10020 and one muramidase LCABL_13470 are annotated as prophage-encoded enzymes and genes encoding holins are present next to the corresponding genes. The identified PGHs can be divided into six classes according to their putative hydrolytic specificities (muramidases, glucosaminidases, amidases, endopeptidases, CHAP-domain-containing enzymes with amidase or endopeptidase activity and carboxypeptidases) predicted on the basis of Pfam domains. Only three of them contain additional domains previously identified as cell-wall binding domains such as LysM [11] or SH3 domains [12].

**Table 1:** The PGH complement of *L. casei* BL23 predicted *in silico* on whole genome sequence.

Locus tag	MM^a^ (kDa)	SP^b^	Putative hydrolytic specificity	Catalytic domain^c^	Other domains^d^
LCABL_02350	100.5	Yes	Muramidase	GH25	
LCABL_12360	28.0	No	Muramidase	GH25	
LCABL_04610	27.4	No	Muramidase	GH25	LysM
LCABL_13470^e^	42.9	Yes	Muramidase	GH25	SH3, LysM
LCABL_12760	24.0	Yes	Glucosaminidase	Glucosaminidase	
LCABL_05960	35.8	Yes	Glucosaminidase and endopeptidase	Glucosaminidase and NplC/P60	
LCABL_17510	46.9	Yes	Amidase	Amidase_3	SH3
LCABL_11280^e^	37.6	No	Amidase	Amidase_2	
LCABL_10020^e^	34.8	No	Amidase	Amidase_5	
LCABL_02770	49.6	Yes	Endopeptidase	NplC/P60	
LCABL_21960	41.4	Yes	Endopeptidase	NplC/P60	
LCABL_00230	42.3	Yes	Endopeptidase or amidase	CHAP	
LCABL_02100	46.9	Yes	Carboxypeptidase	Peptidase_S11	PBP5_C

a Calculated molecular mass.

b SP, signal peptide predicted with SignalP tool [40].

c Catalytic domains were predicted with Pfam domain prediction [41]. Glucosaminidase (PF01832), muramidase (glyco_hydro_25; PF01183), Amidase_2 (PF01510), Amidase_3 (PF01520), CHAP (cysteine, histidine-dependant amidohydrolase/peptidase) domain (amidase or peptidase) (PF05257), NlpC_P60 (PF00877) (including γ-glutamyl-diamino-acid endopeptidases), Peptidase_S11 (PF00768).

d SH3, SH3-domain (PF08460); LysM, LysM-domain (PF01476); PBP5_C (PF07943).

e Putative prophage-encoded PGH.

### Identification of the main PGHs expressed during growth of *L. casei* BL23

By RT-PCR experiments, we showed that the twelve putative PGH genes encoding non-carboxypeptidase-type enzymes are transcribed in *L. casei* BL23 during the exponential growth phase (Figure S1). In order to identify the main PGHs synthesized during exponential phase growth of *L. casei* BL23, we used a proteomic approach combining 1D SDS-PAGE separation and liquid nanochromatography coupled to tandem mass spectrometry (LC-MS/MS) identification of the proteins. Bacterial proteins were fractionated in cell envelope and cytoplasmic fractions and proteins associated non-covalently to the cell wall were extracted with LiCl. Proteins of the different fractions were separated by SDS-PAGE (Figure S2) and analyzed after tryptic digestion by LC-MS/MS. In total, seven PGHs were detected in at least one of the BL23 cell fractions indicating that these PGHs are expressed during bacterial growth (Table 2). The relative amount of each PGH in the different cellular extracts was estimated by calculation of its PAI as previously described [13]. All the identified PGHs appear more abundant in the cell envelope fraction or the LiCl fraction than in the cytoplasmic fraction in agreement with the presence of a signal peptide sequence and according to their presumed cellular functions. On the whole, the putative endopeptidase LCABL_02770 appears as the major PGH in *L. casei* BL23 cell wall. The full-length LCABL_02770 protein is a 494-residue protein with a calculated molecular mass of 49.6 kDa. It exhibits a conserved NlpC/P60 (Pfam00877) domain located in the C-terminal part with a 32-residue N-terminal signal sequence (Table 1).

**Table 2:** Proteomic identification by 1D SDS-PAGE and LC-MS/MS of the PGHs present in *L. casei* BL23 extracts and estimation of relative amounts by PAI calculation.

Protein name^a^	Calculated MM (kDa)	SP^b^	Putative hydrolytic specificity	Protein log(E-value)^c^	PAI values^d^		
					Cytoplasmic extract	Cell envelope extract	LiCl extract
LCABL_02350	100.5	Yes	Muramidase	−196.6	0.91	1.72	0.13
LCABL_02100	46.9	Yes	Carboxypeptidase	−138.9	1.23	2.42	0
LCABL_02770	49.6	Yes	Endopeptidase	−67.0	1.00	0.86	3.86
LCABL_17510	46.9	Yes	Amidase	−67.8	0	0.23	0.73
LCABL_21960	41.4	Yes	Endopeptidase	−26.2	0.18	0.27	0.64
LCABL_00230	42.3	Yes	Endopeptidase or Amidase	−17.5	0.13	0.38	1.00
LCABL_11280	37.6	No	Amidase	−28.0	0	0.40	0

a As defined in Table 1.

b SP, signal peptide predicted with SignalP tool [40].

c Protein log(E-value) is the log of the product of validated unique peptide E-values and was calculated by X!tandem PAPPSO pipeline.

d PAI (Protein Abundance Index) was calculated according to [13] as the number of observed spectra divided by the number of calculated observable peptides and calculated with the X!Tandem pipeline.

Remarkably, LCABL_02770 protein has high amino acid sequence identity (72%) with p75 (Msp1) protein from *Lactobacillus rhamnosus* GG. p75 is one of the major secreted proteins of *L. rhamnosus* GG and was reported to promote the survival and growth of epithelial cells especially under pro-inflammatory conditions [14], [15]. The *L. casei* homolog corresponding to LCABL_02770 (renamed Lc-p75) was previously partially characterized and was shown to be able to cleave PG [16]. However, the exact bond targeted by Lc-p75 was not identified.

### Hydrolytic specificity of Lc-p75 on peptidoglycan

**Figure 1:**
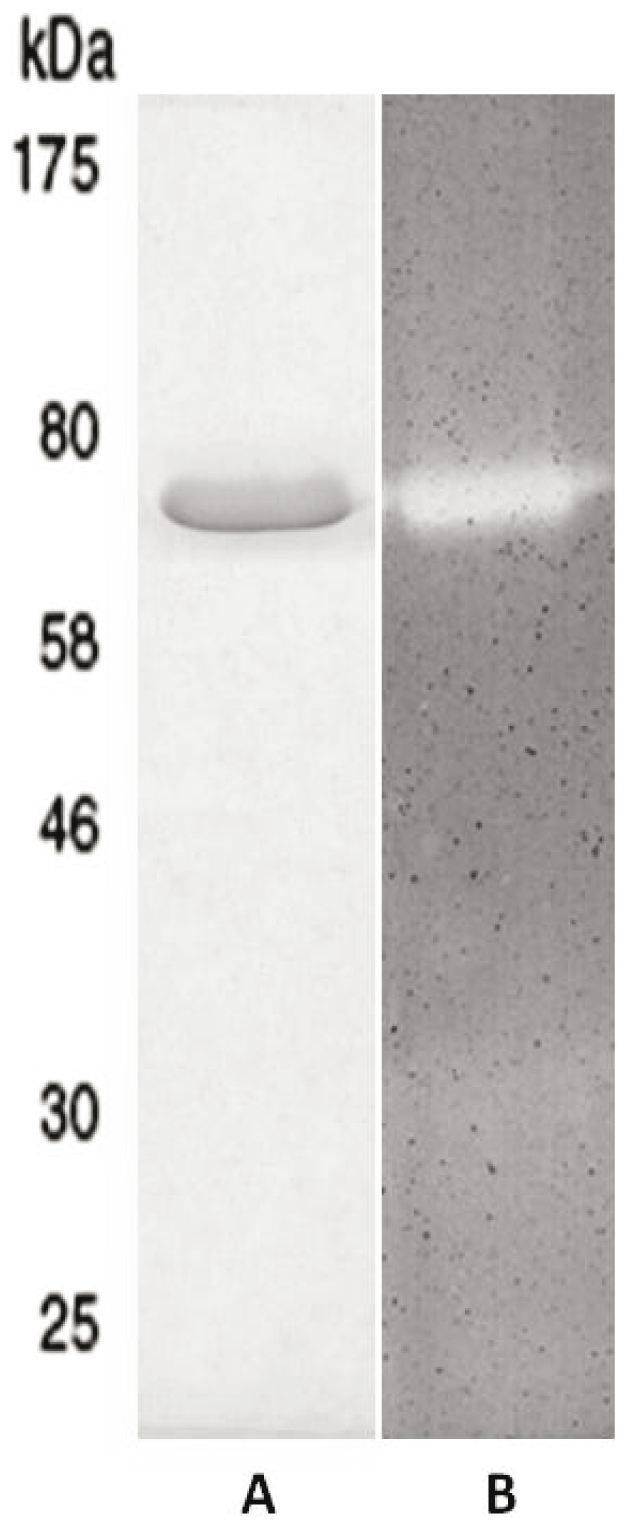
Analysis of purified recombinant Lc-p75. (A) SDS-PAGE after Coomassie blue staining and (B) zymogram assay.

In order to identify Lc-p75 hydrolytic specificity, the protein devoid of signal sequence and with a six-His tag added at its N-terminus was produced in *E. coli* with the use of pBAD plasmid, dedicated to the expression of toxic proteins. The His_6_-tagged protein was purified to homogeneity by nickel-affinity chromatography, followed by anion exchange chromatography (Figure 1). In SDS-PAGE (Figure 1A), purified His_6_-tagged Lc-p75 migrated as a 75-kDa protein whereas its calculated molecular mass is around 50 kDa. The pure protein displayed hydrolytic activity in a zymogram assay (Figure 1B) when *L. casei* cells first treated with TCA were used as substrate. No activity was detected on micrococci in zymogram assay (data not shown).

**Figure 2:**
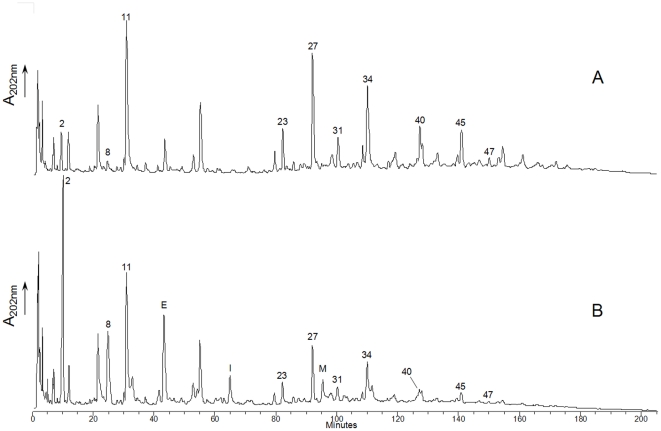
RP-HPLC separation profile of muropeptides obtained from *L. casei* BL23 PG. PG was digested by mutanolysin (A) or by mutanolysin and recombinant Lc-p75 (B). Numbers correspond to the main muropeptides which amounts changed between Panel A and B. Letters indicate new peaks present in Panel B and absent in Panel A. Complete annotation of the chromatograms is presented in Figure S3.

**Figure 3:**
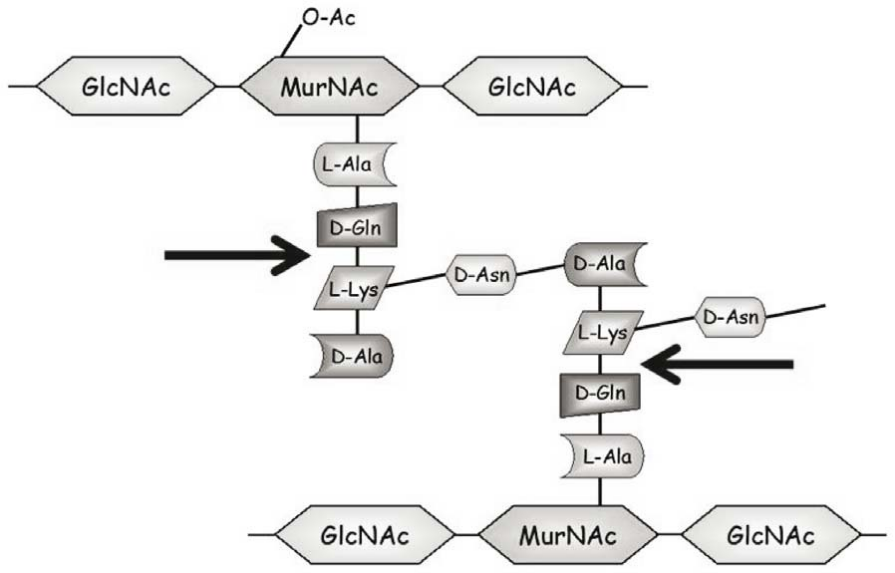
Schematic structure of *L. casei* BL23 peptidoglycan and site of cleavage determined for Lc-p75. GlcNAc, N-acetylglucosamine; MurNAc, N-acetylmuramic acid. MurNAc may be O-acetylated (O-Ac) or not in PG.

We then examined the hydrolytic specificity of recombinant His-tagged purified Lc-p75 on *L. casei* PG first digested with mutanolysin to generate soluble muropeptides. After reduction, half of the muropeptide mixture was incubated with pure His_6_-tagged Lc-p75. Muropeptides obtained after mutanolysin digestion and after mutanolysin plus Lc-p75 digestion were separated by RP-HPLC (Figure 2A, B) and analyzed by Maldi-Tof MS. The structure of 63 muropeptides could be deduced for the reference mutanolysin digest of *L. casei* BL23 PG (Figure 2A, Figure S3 and Table S3). The main changes observed after subsequent Lc-p75 digestion on the muropeptide profile (Figure 2B) are summarized in Table 3. First, after Lc-p75 digestion we observed a large increase (more than 6-fold) of disaccharide dipeptide (Peak 2) and (more than 7-fold) of acetylated dissacharide dipeptide (Peak 8). Concomitantly, a clear decrease of dimers (Peaks 23, 27, 31, 34), trimers (Peaks 40, 45) and tetramers (Peak 47) was observed. In addition, new muropeptides listed in Table 3 (and Table S3) corresponding to partially hydrolyzed muropeptides were identified in the Lc-p75 digest (Figure 2B). These partially hydrolyzed muropeptides arose after the loss of disaccharide dipeptide (mass reduction of 679 Da) or acetylated disaccharide dipeptide (mass reduction of 721 Da) from dimers or trimers present in the mutanolysin digest. As a conclusion, muropeptides generated by Lc-p75 hydrolysis demonstrated that Lc-p75 hydrolyses peptidoglycan bonds inside the stem peptide between D-iGln and L-Lys (Figure 3).

**Table 3:** Main muropeptides from *L. casei* BL23 PG hydrolyzed by Lc-p75 and main products of digestion.

Peak^a^	Proposed structure^b^	Observedm/z	Calculated^c^[M+Na]^+^	% of all peaks^d^ *^,^* e	
				mutanolysin	mutanolysin and Lc-p75
2	Di	720.28	720.29	2.26	15.19
8	Di (Ac)	762.31	762.3	0.79	7.59
11	Tetra-N	1033.44	1033.47	14.12	10.85
E	Tetra-N-(A-K)-N	1346.59	1346.65	ND	6.06
I	Tetra-N-(A-K)-N (Ac)	1388.84	1388.65	ND	2.62
23	Tri-N-Tetra-N	1954.86	1954.89	3.01	1.62
27	Tetra-N-Tetra-N	2025.93	2025.93	9.73	4.26
M	Tetra-N-Tetra-N-(A-K)-N	2339.12	2339.11	ND	1.98
31	Tri-N-Tetra-N (Ac)	1996.68	1996.91	2.70	1.38
34	Tetra-N-Tetra-N (Ac)	2067.94	2067.94	9.18	4.16
40	Tetra-N-Tetra-N-Tetra-N	3018.23	3018.39	3.33	0.89
45	Tetra-N-Tetra-N-Tetra-N (Ac)	3060.42	3060.4	3.83	1.11
47	Tetra-N-Tetra-N-Tetra-N-Tetra-N	4010.44	4010.86	0.77	0.04

a Peak numbers refer to Figure 2 and Figure S3. New peaks obtained after Lc-p75 digestion, are indicated by letters.

b Di, disaccharide dipeptide (L-Ala-D-iGln); Tri, disaccharide tripeptide (L-Ala-D-iGln-L-Lys); Tetra, disaccharide tetrapeptide (L-Ala-D-iGln-L-Lys-D-Ala); Disaccharide, GlcNAc-MurNAc; Ac, acetylation on MurNAc, iGln, isoglutamine; N, D-Asn; A, D-Ala; K, L-Lys.

c Sodiated molecular ions were the most abundant ones on MALDI-TOF mass spectra for all muropeptides.

d Percentage of each peak was calculated as the ratio of the peak area over the sum of areas of all the peaks identified in the corresponding chromatogram (see Table S3).

e ND, non detected.

In order to investigate in more details the specificity of Lc-p75, pure isolated muropeptides were digested with the purified recombinant Lc-p75 (Table 4). The results show first that multimeric muropeptides are better substrates than monomers. Regarding monomers, we can conclude that muropeptides with tetrapeptide stem peptides are better substrates than muropeptides with tripeptide chains which are very poor substrates. The presence of an Asn bridge as well as MurNAc O-acetylation tends to increase Lc-p75 activity. All these results identified Lc-p75 as a γ-D-glutamyl-L-lysyl-endopeptidase.

**Table 4:** Lc-p75 activity on purified muropeptides selected as substrates.

Muropeptide substrate^a^^,^ b	Undigested muropeptide(%)^c^	Di^a^(%)^c^	Other forms(%)^c^ *^,^* d
Tri	98.1	1.9	0
Tri-N	98.6	1.4	0
Tri-N (Ac)	99.4	0.6	0
Tetra	61.1	38.9	0
Tetra-N	77.4	22.6	0
Tetra-N (Ac)	15.2	84.7	0
Tri-N-Tetra-N	0	33.7	66.3
Tri-N-Tetra-N (Ac)	0	37.8	62.2
Tetra-N-Tetra-N	0	80.3	19.7
Tetra-N-Tetra-N (Ac)	0	86.2	13.7
Tetra-N-Tetra-N (2Ac)	0	94.4	5.6
Tetra-N-Tetra-N-Tetra-N	0	80.6	19.4
Tetra-N-Tetra-N-Tetra-N (Ac)	0	95.0	4.9
Tetra-N-Tetra-N-Tetra-N (2Ac)	0	95.4	4.6

a Di, disaccharide dipeptide; Tri, disaccharide tripeptide (L-Ala-D-iGln-L-Lys); Tetra, disaccharide tetrapeptide (L-Ala-D-iGln-L-Lys-D-Ala); Disaccharide, GlcNAc-MurNAc; iGln, isoglutamine; N, Asn; Ac, acetylation on MurNAc.

b Similar amounts of each muropeptide were used for each test.

c Percentage of each peak was calculated as the ratio of the peak area over the sum of areas of all the peaks identified in the corresponding chromatogram.

d Other forms of muropeptides resulting from partial digestion of the substrate.

### Immunolocalization of Lc-p75

**Figure 4:**
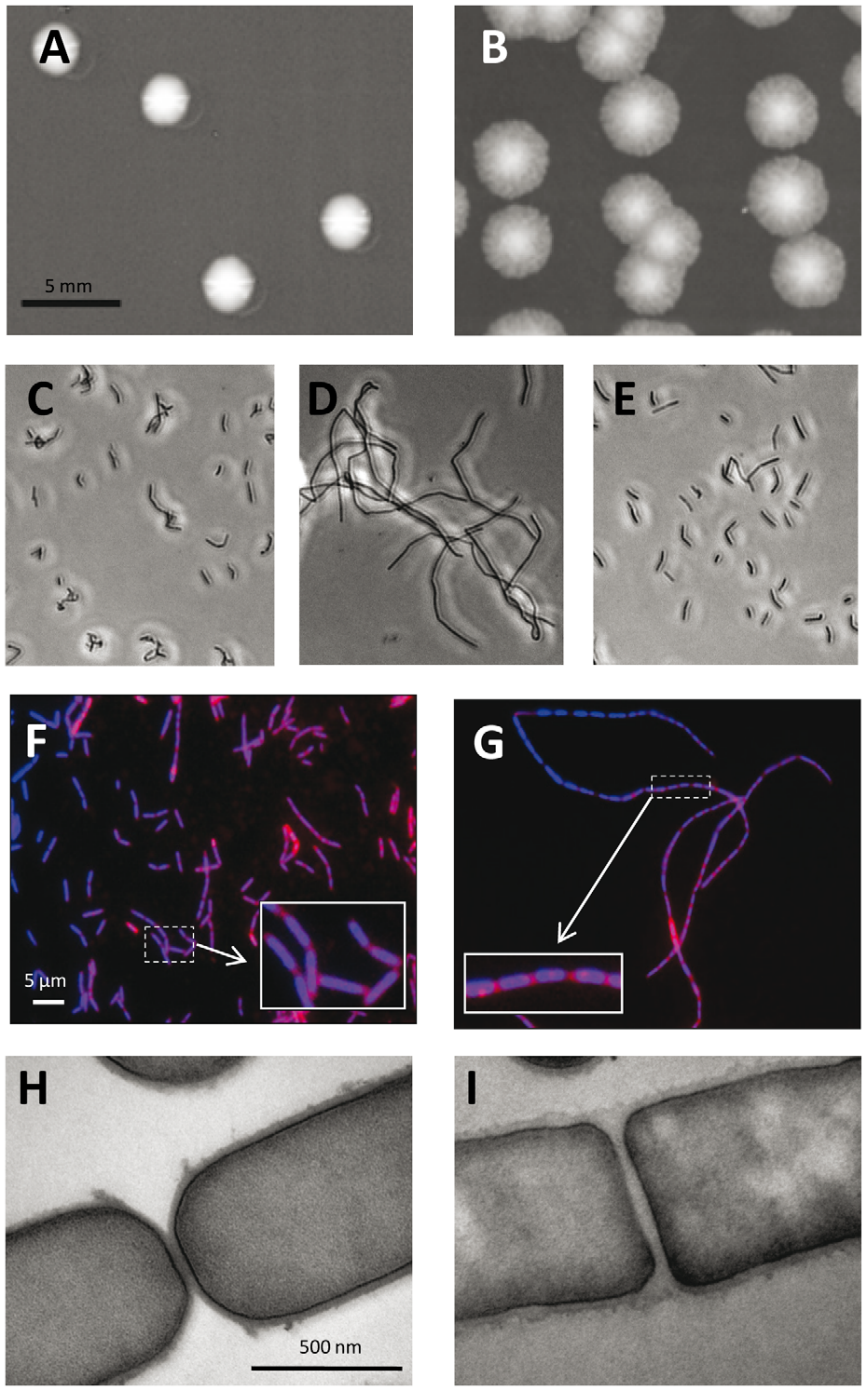
Phenotype comparison between wild type *L. casei* BL23, Lc-p75-negative mutant and complemented Lc-p75 mutant. Pictures of wild type *L. casei* (A, C, F, H), Lc-p75-negative mutant (B, D, I) and complemented Lc-p75 mutant (E). Colony morphology (A, B), phase contrast microscopy (C, D, E) fluorescence microscopy with merged FM-4-64 (red) and DAPI (blue) staining (F, G) and transmission electron microscopy (H, I).

The gene *lcabl_02270* encoding Lc-p75 was previously inactivated by single cross-over in strain *L. casei* BL23 and the resulting mutant was shown to form long chains [16]. We confirmed the role of Lc-p75 in daughter cell separation in *L. casei* BL23 by constructing a double-cross over (DCO) mutant by a method previously established in *L. plantarum* [17], [18]. First observations of the morphology of isolated colonies on MRS agar plates indicated that the DCO negative mutant formed bigger colonies with non regular shape and jagged border compared to the wild type strain BL23 (Figure 4A, B). In liquid broth, the mutant displayed a fast-sedimenting phenotype linked to the formation of long chains (Fig. 4C, D). Labeling of Lc-p75 DCO mutant cells with DAPI and the membrane dye FM4–64 showed that the chains are made of unseparated cells (Figure 4F, G). TEM observations indicate that in the mutant strain, septa are not digested and cell separation is blocked (Figure 4H, I). Complemented Lc-p75 mutant was able to recover the wild type short-chain phenotype (Fig. 4E).

**Figure 5:**
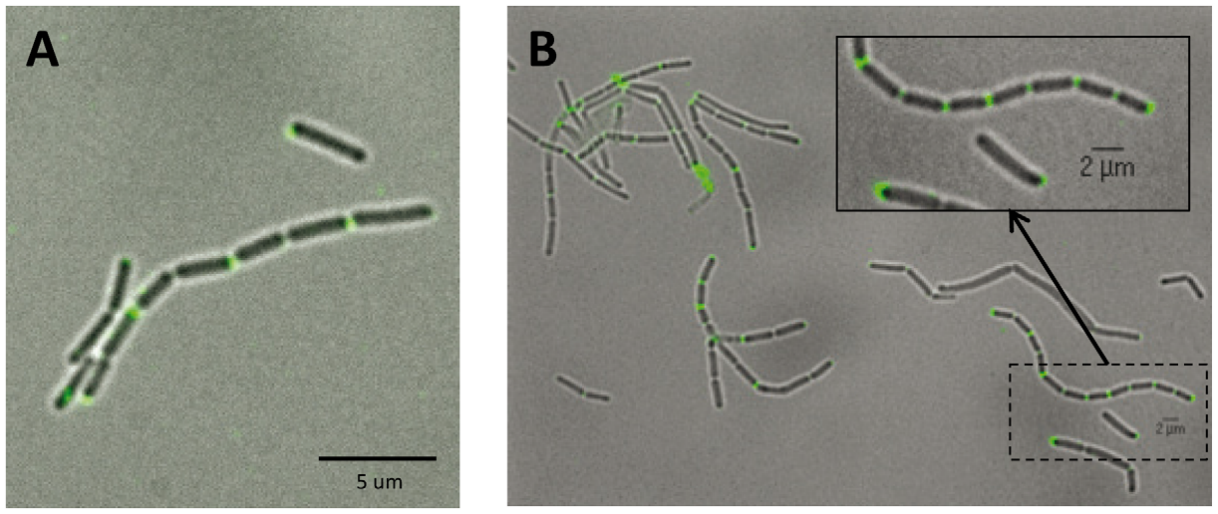
Indirect immunofluorescence localization of Strep-tagged Lc-p75 in *L. casei*. Strep-tagged Lc-p75 was localized in overexpressing strain (A) and in complemented negative mutant (B) with monoclonal antibody directed against Strep-tag as first antibody.

To gain further insights in the role of Lc2270 in cell separation, we localized Lc-p75 by indirect immunofluorescence experiments. Strep-tagged Lc-p75 protein was expressed under the control of the nisin-inducible promoter in wild-type BL23 and in Lc-p75-negative DCO mutant. We checked that Strep-tagged Lc-p75 was produced upon nisin induction in both strains (Figure S4 and data not shown). As it can be observed on Fig. 5, Strep-tagged Lc-p75 complements the long chain phenotype of the mutant leading to short chains (Fig. 5B) like the wild-type strain expressing Strep-tagged Lc-p75 (Fig. 5A); also loss of the fast-sedimenting phenotype was observed (data not shown). Using a specific monoclonal antibody directed against Strep-tag, we were able to localize Strep-tagged Lc-p75 at the septa in the wild type strain as well as in the negative mutant (Figure 5A, B). As a control, no fluorescence was detected in wild type BL23 strain (data not shown).

### Lc-p75 is secreted as an active PGH

**Figure 6:**
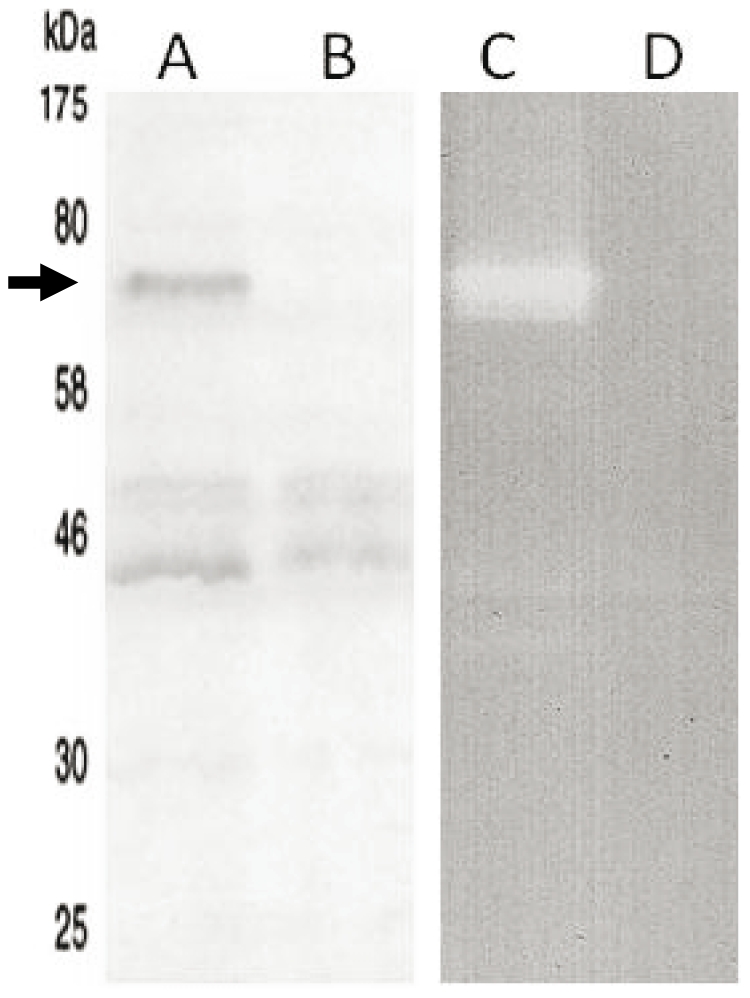
Detection of Lc-p75 in culture supernatant. Analysis by SDS-PAGE (A, B) and zymogram assay (C, D) of the culture supernatant of wild type BL23 (A, C) and negative mutant (B, D) grown on AOAC medium. Lc-p75 is indicated by an arrow.

*L. casei* Lc-p75 was previously reported to be secreted in the culture supernatant like its *L. rhamnosus* p75 (Msp1) homolog [16]. In order to examine whether Lc-p75 is active after secretion, *L. casei* BL23 and its derivative Lc-p75-negative mutant were grown in AOAC broth and 60-fold concentrated supernatants were analyzed by SDS-PAGE and zymogram. As shown on Figure 6 (lanes A, B), one of the major stained bands migrating at 75 kDa, detected in wild type BL23 and absent in the mutant strain was identified as Lc-p75 by peptide mass fingerprint (data not shown). In zymogram assay with TCA-treated *L. casei* cells as substrate (Figure 6, lanes C, D), an activity band was visualized around 75 kDa in BL23 and absent in the mutant. These results indicate that Lc-p75 is active after secretion in the culture supernatant of *L. casei* BL23.

### Effect of Lc-p75 inactivation on *L. casei* PG structure

PG was extracted from wild type BL23 and Lc-p75-negative DCO mutant, digested with mutanolysin and the muropeptides were separated by RP-HPLC. Comparison of the RP-HPLC muropeptide profiles obtained for the two strains (data not shown) revealed that *lcabl_02770* inactivation leads to a decrease of the amount of disaccharide peptide and of acetylated disaccharide dipeptide. The total amount of disaccharide dipeptide with acetylation or not constitutes 6.3% of the total muropeptides in wild type BL23 whereas it constitutes 5.4% in the Lc-p75-negative mutant. These results suggest that Lc-p75 is active inside the cell wall in living cells and is able to modulate the amount of constituent disaccharide dipeptide in whole PG.

## Discussion

The PGH complement of *L. casei* BL23 was first determined *in silico*. As well as the three prophage-encoded lysins, ten putative PGHs were detected. The *L. casei* PGH complement comprises enzymes of various specificities but no lytic transglycosylase. Thanks to a proteomic analysis based on LC-MS/MS analysis, we identified the most abundant PGHs expressed during growth of *L. casei* BL23. The major PGH expressed in the cell wall was a putative endopeptidase named Lc-p75, with a catalytic domain of the NlpC/P60 family.

Lc-p75 is the homolog of p75 (Msp1) protein, which is one of the major proteins secreted by the probiotic *L. rhamnosus* GG. Previous studies have shown that p75 (Msp1) purified from *L. rhamnosus* GG supernatant prevents cytokine-induced apoptosis in intestinal epithelial cells and promote intestinal homeostasis [14], [15]. The *L. casei* BL23 homolog was shown previously to be able to hydrolyze PG-derived muropeptides [16]. In this study, we identified the cleavage site of Lc-p75 inside PG and demonstrated that this enzyme is a γ-D-glutamyl-L-lysyl-endopeptidase. We observed that tetrapeptide chains are better substrates than tripeptide chains in muropeptides. In addition, we showed that it hydrolyzes preferentially multimeric muropeptides rather than monomeric ones. We detected Lc-p75 activity in zymogram assay, but only when *L. casei* cells were previously treated with TCA suggesting that a cell wall polymer such as polysaccharide should be removed from the bacterial cell wall in order to make PG accessible for sufficient hydrolysis and appearance of a clear band in the gel. Several cell wall-associated polysaccharides were described in *L. casei* in a previous report [19]. Noteworthy, the p75 (Msp1) protein of *L. rhamnosus* was recently found to be equally a cell wall hydrolase with a γ-D-glutamyl-L-lysyl-endopeptidase specificity [20].

Inactivation of *lcabl_02770* gene leads to the formation of long chains as described previously [16] and in this study. We show here that the long-chain phenotype was reversed upon mutant complementation. In addition, we confirmed by FM4-64 membrane labelling and TEM that cell separation was impaired in the mutant strain because septa were not digested. Lc-p75 constitutes a new example of the structural diversity of PGHs involved in daughter cell separation in Firmicutes [21]. Other enzymes with NlpC/P60 catalytic domain were previously reported to perform this function but with different domain structures; in *L. casei* Lc-p75, no known cell wall binding domain could be identified.

By immunofluorescence experiments, we showed that Lc-p75 is located at the level of bacterial septa when associated to the cell wall. In addition, Lc-p75 is one of the major secreted proteins by *L. casei* BL23 and it is active after secretion into culture supernatant as shown by zymogram assay. We hypothesize that as reported previously for the major *L. lactis* autolysin AcmA, a cell wall component is hindering Lc-p75 binding sites at the bacterial surface and these sites would be accessible only at the cell poles [11]. Since the conserved catalytic domain is located at the C-terminus of Lc-p75, the N-terminal part is candidate for being involved in cell wall binding. However, none of the known cell-wall-binding domains, or other conserved characterized domain was found at the N-terminal part of Lc-p75. Noteworthy, the N-terminal domain is conserved in *L. rhamnosus* p75 (Msp1) although presenting less sequence identity than the catalytic domain.

PG or PG-derived fragments have been previously shown to be microbial-associated molecular patterns (MAMPS) sensed by host pattern-recognition receptors (PRRs) such as Toll-like receptors (TLRs) [22] or NOD-like receptors (NLRs) [23]. These receptors signal the bacterial presence to the host and can activate a cascade of signals leading to immune response against pathogens. PGH activity can promote the release of muropeptides into the host or host immune cells [24], [25], [26]. In the case of commensal or probiotic bacteria, the underlying molecular mechanisms and the signalling pathways involved in bacteria-host cross-talk are still poorly identified. Nevertheless, recent studies conducted with lactobacilli strains highlighted the role of PG and PG-derived fragments in the modulation of cytokine production by macrophages or dendritic cells through the involvement of PRRs such as TLR2 and NOD2 [8], [9]. In *L. casei* BL23, PG structural analysis of the negative mutant indicated that Lc-p75 endopeptidase activity inside bacterial cell wall is able to modulate the amount of disaccharide dipeptide muropeptide present in PG. Since disaccharide dipeptide contains muramyl-dipeptide sensed by NOD2 receptors, our results suggest that wild type and mutant strains could interact differently with host cells expressing NOD2 receptors. In a recent study, the anti-inflammatory capacity of a *Lactobacillus* strain was shown to be linked to the presence of a muropeptide (disaccharide tripeptide) which is a NOD2 activator, whereas this muropeptide was absent in a *Lactobacillus* strain devoid of anti-inflammatory properties [9].

As a conclusion, Lc-p75 plays a key role in bacterial physiology, since among the ten identified PGHs, not encoded by prophages, it has a major role in daughter cell separation after cell division. Further work will aim at evaluating its involvement in the anti-inflammatory properties of *L. casei* BL23 and in the cross talk between *L. casei* BL23 and host epithelial and dendritic cells. In the light of our results, it will be necessary to consider both its role as a secreted protein that can interact with host cell receptors and its role as a PGH able to release muropeptides that can be sensed by other receptors.

## Materials and Methods

### Bacterial strains, plasmids, and growth conditions

The bacterial strains and plasmids used in this study are listed in Table S1. *Escherichia coli* strains were grown in Luria–Bertani (LB) medium with shaking at 37°C. *L. casei* strains were routinely grown in MRS broth or when indicated in AOAC medium (Difco Laboratories) at 37°C. When required, antibiotic concentrations used were 150 µg/ml erythromycin, 10 µg/ml chloramphenicol or 100 µg/ml ampicillin for *E. coli* and 5 µg/ml erythromycin or 5 µg/ml chloramphenicol for *L. casei*. Growth was monitored by optical density (OD) measurement at 600 nm (OD_600_) with a spectrophotometer (Spectronic 20, Genesys).

### Protein identification in bacterial extracts by 1D SDS-PAGE and LC-MS/MS

Protein identification by 1D SDS-PAGE and LC-MS/MS was performed as described previously [27] by the Plateforme d'Analyse Protéomique de Paris Sud Ouest (PAPSSO) (INRA, Jouy-en-Josas, France). Briefly, cells were harvested during logarithmic growth phase by centrifugation and disrupted using Bazic Z cell disruptor (Constant Systems Ltd) at a pressure of 2500 bar. Unbroken cells were removed by centrifugation at 4000× g during 15 min at 4°C. Then the supernatant was centrifuged at 220,000× g for 30 min at 4°C to separate « cell envelope fraction » from cytosolic proteins. Also, cell wall-associated proteins were extracted by incubation of *L. casei* cells during 30 min at 4°C by 4 M LiCl in 20 mM Tris-HCl, pH 7. Ten micrograms of proteins from cytosolic and cell envelope extracts and around one µg of proteins from LiCl-extract were separated by 4–12% gradient SDS-PAGE (Invitrogen) and stain with Blue safe stain (Invitrogen). The gel was washed with deionized H_2_O and cut horizontally into 26 sections lane by lane. Gel slices were washed, reduced with DTT, alkylated with iodoacetamide, dried and incubated overnight at 37°C with 125 ng sequencing grade trypsin (Promega) as described previously [27]. Each tryptic digest was analyzed by liquid chromatography coupled to tandem mass spectrometry (LC-MS/MS). Analysis were performed on an Ultimate 3000 LC system (Dionex) connected to a LTQ-Orbitrap Discovery mass spectrometer (Thermo Fisher). Protein identification was performed using X!tandem software (X!Tandem tornado 2008.02.01.3, http://www.thegpm.org) against a protein database of BL23 strain (GenBank: NC_010999.1) associated to a classical contaminant database as described in Text S1.

The relative abundance of each detected PGH was estimated by calculation of its Protein Abundance Index (PAI) as previously described [13], [28] after extraction of the PGH proteins to the list of validated proteins (1485 proteins after elimination of contaminant proteins). For PAI calculation, observable tryptic peptides were taken to be those in the mass range 800 to 2500 Da. It was calculated with the X!tandem parser developed and used on PAPPSO platform (X!Tandem pipeline version 3.1.2 http://pappso.inra.fr/bioinfo/xtandempipeline/).

### DNA techniques and electrotransformation

General molecular biology techniques were used as previously described [29]. Electro-transformation of *E. coli* was performed according to [30]. Electrocompetent *L. casei* cells were prepared as previously described [31]. PCR were performed with the Phusion high-fidelity DNA polymerase (Finnzymes) using a Mastercycler gradient thermocycler (Eppendorf). The primers used in this study were purchased from Eurogentec and they are listed in Table S2. Cloning of DNA fragments in plasmids pNZ5319 and pMSP3545 was initially performed in *E. coli* TG1 repA+, then the recombinant positive plasmids were extracted using QIAprep Spin Miniprep kit (Qiagen) and subsequently transferred to *L. casei* BL23 by electroporation using a Gene-Pulser apparatus (Bio-Rad).

### RT-PCR experiments

Transcription of the *in silico* detected PGH genes in *L. casei* BL23 was tested by RT-PCR experiments. Total RNA was extracted using the TRIzol Reagent (Invitrogen) and cDNA synthesis was performed from 1 µg of RNA with Moloney murine leukemia virus reverse transcriptase (Invitrogen) according to the manufacturer's instructions. Primers located inside the different genes, were designed using Primer 3 software (http://frodo.wi.mit.edu/primer3/) and are listed in Table S4. PCR was performed as described above. The *L. casei tuf* gene, encoding the elongation factor TU, was used as a positive control in RT-PCR experiments as described previously [32]. The absence of contamination of RNA samples by genomic DNA was checked by performing PCR with the *tuf*-specific primers on total RNA preparation.

### Construction of the *lcabl_02770* deletion mutant

Construction of the *L. casei* BL23 gene deletion mutant for *lcabl_02770* gene was performed using the *cre*-*lox* based system described previously for *L. plantarum* [17], [18]. This system allows replacement by double crossover (DCO) of the target gene by a chloramphenicol resistance cassette flanked by two *lox* sites containing mutations within the inverted repeats (*lox66*-P_32_ *cat*-*lox71*). Briefly, the upstream and downstream flanking regions of the target gene were amplified by PCR using *L. casei* chromosomal DNA as template and primers listed in Table S2. Subsequently, PCR products were cloned into the SwaI and SmaI sites respectively, of the non-replicating integration vector pNZ5319 [17]. The recombinant mutagenesis plasmid was extracted from *E. coli* and transformed into competent *L. casei* BL23 cells. Colonies displaying a chloramphenicol-resistant and erythromycin-sensitive phenotype represent candidates with DCO gene replacement. Selected colonies were checked by PCR with primers flanking the sites of recombination to confirm *cat*-replacement genotype. Subsequently, the *lox66*-P_32_ *cat*-*lox71* cassette was excised by expression of the *cre* recombinase as described [17] using the thermosensitive *cre* expression plasmid pGhostcre [33] transformed previously into the selected double crossover mutants. To cure pGhost plasmid, bacteria were grown at 42°C in the absence of antibiotics until erythromycin sensitive clones were found. Clones displaying the expected erythromycin- and chloramphenicol-sensitive phenotype were checked for Cre-mediated recombination and correct excision of the chloramphenicol cassette, with primers flanking the recombination locus (Table S2). One clone was selected for further study.

### Cloning and overexpression of *lcabl_02770* and *lcabl_02770* with Strep-tag in *L. casei*

Plasmid pMSP3545 [34] which carries both the *nisA* inducible promoter and the *nisRK* genes required for nisin-controlled gene expression [35] was used as expression vector in *L. casei* BL23. The *lcabl_02770* gene was amplified by PCR using the primer pair 2770-pMSP-F and 2770-pBAD-R (Table S2). Also, a construct was made to obtain Lc-p75 fused at its C-terminus with Strep-tagII sequence (Trp-Ser-His-Pro-Gln-Phe-Glu-Lys). Strep-tag was added by PCR by amplifying *lcabl_02770* gene with a reverse primer encoding the Strep-tagII sequence (Table S2). The amplified fragments were digested with NcoI and XbaI and ligated to NcoI/XbaI restricted pMSP3545 plasmid. The ligation mixtures were electroporated into *E. coli* and recombinant plasmids with an insert, named pMSP::2770 and pMSP::2770StrepTag, were isolated. They were then transformed in *L. casei* BL23. To induce the expression of the genes under the control of the *nisA* expression signals, nisin A (Sigma-Aldrich) was used at 50 ng/ml final concentration.

### SDS-PAGE and zymogram

SDS-PAGE was performed with 10% (w/v) polyacrylamide separating gels. Gels were stained with PageBlue Protein Staining Solution (Fermentas). Zymogram was used to detect cell wall hydrolase activity and was performed as described previously [36]. *L. casei* cells used as enzyme substrate were previously treated with 10% TCA during 10 min at 100°C, washed 3 times with cold PBS and 3 times with deionized water. The TCA-treated *L. casei* cells were included at 0.4% (w/v) into polyacrylamide gels. After sample migration, the gels were washed three times for 15 min in deionized water at room temperature and then incubated in 50 mM sodium phosphate buffer, pH 6.5, containing 1 mM DTT, and 0.1% (v/v) Triton X-100 overnight at 37°C. Stained polyacrylamide gels and zymogram gels were digitized with a DuoScan T1200 scanner (Agfa).

### Expression in *E. coli* and purification of His-tagged protein

His_6_-tagged Lc-p75 devoid of its putative signal sequence was overexpressed in *E. coli* TOP10 with pBAD/His B expression vector. Briefly, *lcabl_02770* without its 5′ region was amplified by PCR from *L. casei* BL23 DNA using primers listed in Table S2. The PCR fragment was cloned into XhoI and HindIII restriction sites of pBAD/His B vector. Expression was induced with L-arabinose at 0.2% final concentration according to manufacturer's instructions (Invitrogen). Bacteria were harvested 4 h after addition of arabinose by centrifugation and disrupted using BAZIC Z cell disruptor (Constant Systems Ltd) at a pressure of 1600 bar. The clarified soluble fraction containing the recombinant protein was loaded onto a HisTrap HP 1 ml-column (GE Healthcare) connected to a FPLC AKTA chromatography system (GE Healthcare). His_6_-tagged Lc-p75 was eluted using a linear gradient of imidazole from 30 mM to 400 mM. Fractions containing His_6_-tagged Lc-p75 were combined and desalted by Fast Desalting HR 10/10 column (GE Healthcare). Anion exchange chromatography was selected as a polishing step. Desalted sample was loaded onto Mono Q 5/50 GL column (GE Healthcare) and His_6_-tagged Lc-p75 was eluted using a linear gradient of NaCl from 0 to 250 mM in Tris-HCl 20 mM pH 8.0. The purity of the His-tagged protein was checked by SDS-PAGE. The pure protein was then concentrated by ultrafiltration with an Amicon Ultra-4 Centrifugal Filter Units with 30 kDa cut-off (Millipore). Protein concentration was measured using BCA method (BCA Protein Assay Kit, Thermo Scientific). The pure protein was stored at −20°C.

### Peptidoglycan extraction and structural analysis

PG was extracted from *L. casei* BL23 cells according to the protocol described previously for *L. lactis* [37], [38] with some modifications. Additional DNase (50 µg/ml) and RNase (50 µg/ml) treatments were applied before hydrofluoric acid treatment. The material was lyophilized and then stored at −20°C. Purified PG (2 mg dry weight) was digested with mutanolysin from *Streptomyces globisporus* (Sigma) (2500 U/ml) for 19 h in 25 mM sodium phosphate buffer at 37°C. The resulting soluble muropeptides were reduced with sodium borohydride. They were separated by reverse phase-high-pressure liquid chromatography (RP-HPLC) with a Nucleodur C18 Pyramid column (150/3 mm; particle size, 3 µm) (Macherey-Nagel) at 50°C using ammonium phosphate buffer and methanol linear gradient [37]. Muropeptides were analyzed without desalting by MALDI-TOF mass spectrometry (MS) using a Voyager-DE STR mass spectrometer (Applied Biosystems) as reported previously [37].

### Determination of the hydrolytic bond specificity of Lc-p75 on peptidoglycan

Purified PG (2 mg dry weight) was first digested with mutanolysin as described above and the resulting soluble muropeptides were reduced with sodium borohydride. The mixture was adjusted to pH 6.5 and supplemented with 5 µM DTT. To determine the hydrolytic specificity of Lc-p75, 80 µg of pure His_6_-tagged Lc-p75 was incubated with 200 µl of the muropeptide mixture at 37°C during 24 h. Control sample was incubated in the same conditions without Lc-p75. The samples were boiled for 3 min and the products were separated by reverse phase high pressure liquid chromatography (RP-HPLC) and analyzed by MALDI-TOF MS as described above.

The specificity of Lc-p75 was also examined on purified muropeptides. Selected *L. casei* BL23 muropeptides obtained after mutanolysin digest were collected after RP-HPLC separation and desalted on a Betasil C_18_ column (4.6×250 mm, Thermo Electron Corporation) with acetonitrile/formic acid buffer system and dried with speed-vacuum. Similar amounts of each purified muropeptide, estimated according to the corresponding peak areas, were then incubated with 10 µg of purified His_6_-tagged Lc-p75 in a final volume of 50 µl in the conditions described above. The reaction products were then analyzed by RP-HPLC and MALDI-TOF MS as described above.

### Microscopy

Microscopy images were taken with a phase contrast microscope Leica DM1000 equipped with a Topview 1.3MP camera (Motic). Fluorescent dyes, DAPI and the membrane stain FM4-64 (Invitrogen), were used for wild type and negative mutant staining and pictures were taken with Leica DMRA2 microscope connected to CCD camera (Roper). Overlays images were generated with the MetaMorph software. Transmission electron microscopy (TEM) was performed after inclusion of bacteria in Epon on thin sections as described previously [39] by the MIMA2 platform (INRA, Jouy-en-Josas, France).

### Immunofluorescence microscopy

Indirect immunofluorescence was used to determine cellular localization of Lc-p75 in *L. casei* BL23 cells. The Lc-p75-negative mutant and the wild-type BL23 strain complemented with Strep-tagged Lc-p75 were grown in 10 ml MRS broth with 50 ng/ml nisin to induce expression of the tagged-Lc-p75. Bacteria were harvested at OD 0.2 and washed twice with cold PBS. They were incubated in PBS containing 2% BSA for 15 min, then with a monoclonal antibody directed against StrepTagII (IBA GmbH) (10 µg/ml) for 1 h at room temperature. After 3 washes with PBS, goat anti-mouse FITC-conjugate (Sigma Aldrich) diluted 1∶40 in PBS containing 2% BSA, was added for 1 h at room temperature in the dark. Bacteria were washed another 3 times with PBS. Cells were immobilized on microscope slides covered with a thin film of 1.5% agarose in H_2_O, then examined by epifluorescence microscopy with a Leica DMRA2 microscope. Images were acquired by using a charge-coupled device camera. Images were analyzed with the supplied MethaMorph software making overlays of bright field and fluorescent images.

## Supporting Information

Text S1 **Protein identification by MS-MS analysis with X!tandem software.**
(PDF)

Figure S1 **Detection by RT-PCR of the transcripts corresponding to the** ***in silico*** **detected PGHs in** ***L. casei*** **BL23.** Elongation factor TU (tuf gene) chosen as a positive control. (A) Control PCR experiments on *L. casei* BL23 genomic DNA with the different primer pairs selected for each PGH gene and the *tuf* gene; (B) RT-PCR experiments with the same primer pairs on total RNA extracted from exponential phase culture; (C) PCR with the *tuf*-specific primers on total RNA as a negative control.
(TIF)

Figure S2 **SDS-PAGE of proteins from cytoplasmic (A) cell envelope (B) and lithium chloride (C) fractions prepared from** ***L. casei*** **BL23.** The gel was stained with colloidal Coomasie blue. Labeled gel sections from 1 to 26 were cut lane by lane, then digested with trypsin and analyzed separately by LC-MS/MS.
(TIF)

Figure S3 **RP-HPLC profile of muropeptides obtained from** ***L. casei*** **BL23 PG digested by mutanolysin (A) or by mutanolysin and recombinant Lc-p75 (B).**
(PDF)

Figure S4 **Detection of Strep-tagged Lc-p75 in a culture supernatant of the** ***lcabl_02770*** **-negative mutant (PAR006).** (A) SDS-PAGE gel, (B) Western Blot with monoclonal antibody directed against Strep-tag and (C) zymogram.
(TIF)

Table S1 **Bacterial strains and plasmids.**
(PDF)

Table S2 **Primers used for cloning and validation.**
(PDF)

Table S3 **Structures, molecular masses and proportions of muropeptides obtained from** ***L. casei*** **BL23 PG digested by mutanolysin or by mutanolysin and recombinant Lc-p75.**
(PDF)

Table S4 **Primers used for RT-PCR experiments.**
(PDF)
