# Supplementary material for: Analysis of the Peptidoglycan Hydrolase Complement of Lactobacillus casei and Characterization of the Major γ-D-Glutamyl-L-Lysyl-Endopeptidase
Source: PLoS One. 2012 Feb 27;7(2):e32301. doi: 10.1371/journal.pone.0032301 (PMC3288076; doi:10.1371/journal.pone.0032301)
Supplement: Text S1 — Protein identification by MS-MS analysis with X!tandem software. (PDF) [file pone.0032301.s001.pdf]

Supporting information S1

Protein identification by MS-MS analysis with X!tandem software

The raw data produced on LTQ-Orbitrap mass spectrometer were first converted in mzXML file with ReADW (http://sashimi.sourceforge.net) and in a second time the proteins identification was performed. The X!Tandem search parameters for the first pass were: trypsin specificity, with one missed cleavage, fixed alkylation of cysteine and variable oxidation of methionine. The mass tolerance was fixed to 10 ppm for precursor ions and 0.5 Da for fragment ions. In the first pass the identification of peptide was accepted if their E value $\geq$ 0.1. We performed a second pass called 'data refinement' on the previous identified proteins which included to the list of the identified sequences, the semi tryptic peptide with N-terminal acetylation and the same set of parameters for cysteine and methionine modifications. In the data refine mode, the identification of peptide was accepted if their E value $\geq$ 0.01. All xml file results for each gel band were merged and parsed with the X!tandem pipeline developed on PAPPSO (version 3.1.2, http://pappso.inra.fr/bioinfo/xtandempipeline/) with a multiple threshold filter applied at the protein level and consisting of a protein *E*-value of 10^{-4} validated with a minimum of two different peptide sequences, in at least one piece of gel, with a peptide *E*-value lower than 0.05. The level of False Discovery Rate (FDR) value was calculated with the Xtandem pipeline against a protein reverse database. The FDR was estimated to be 0% and 0.07 % for proteins and peptides respectively.
