# Supplementary figures and images for: Analysis of the Peptidoglycan Hydrolase Complement of Lactobacillus casei and Characterization of the Major γ-D-Glutamyl-L-Lysyl-Endopeptidase
Source: PLoS One. 2012 Feb 27;7(2):e32301. doi: 10.1371/journal.pone.0032301 (PMC3288076; doi:10.1371/journal.pone.0032301)

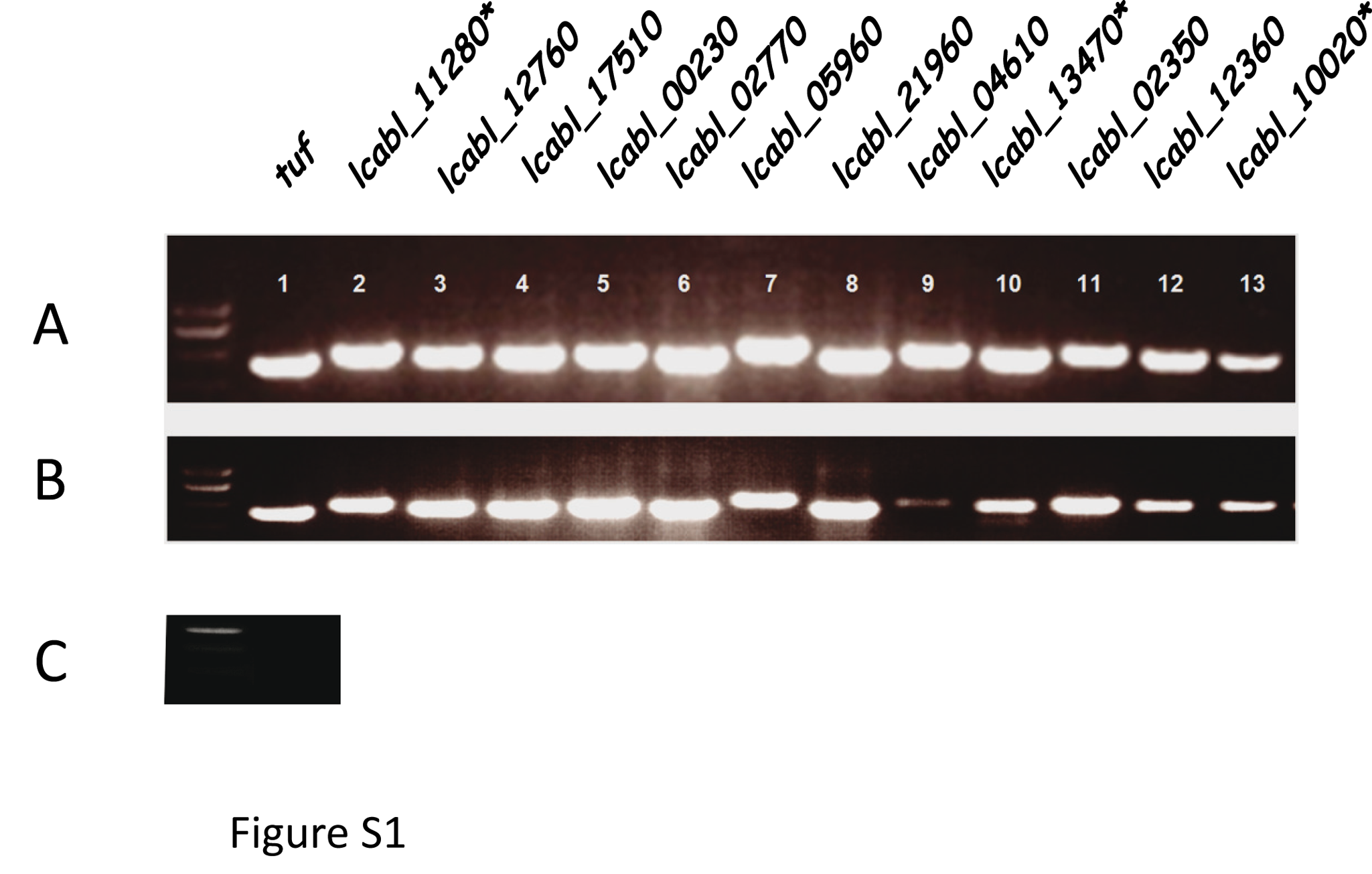

Supplement: Figure S1 — Detection by RT-PCR of the transcripts corresponding to the in silico detected PGHs in L. casei BL23. Elongation factor TU (tuf gene) chosen as a positive control. (A) Control PCR experiments on L. casei BL23 genomic DNA with the different primer pairs selected for each PGH gene and the tuf gene; (B) RT-PCR experiments with the same primer pairs on total RNA extracted from exponential phase culture; (C) PCR with the tuf-specific primers on total RNA as a negative control. (TIF) [file pone.0032301.s002.tif]

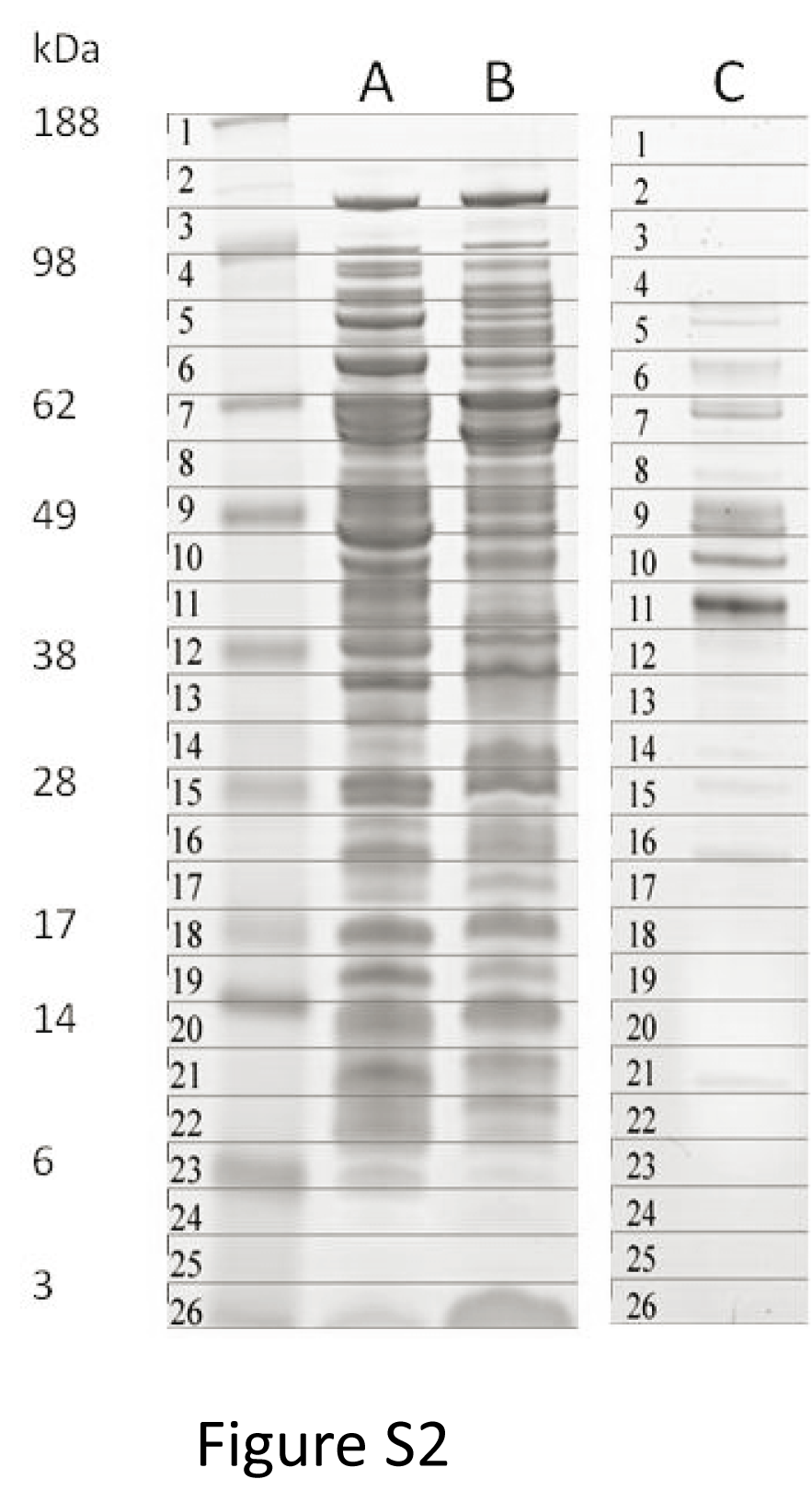

Supplement: Figure S2 — SDS-PAGE of proteins from cytoplasmic (A) cell envelope (B) and lithium chloride (C) fractions prepared from L. casei BL23. The gel was stained with colloidal Coomasie blue. Labeled gel sections from 1 to 26 were cut lane by lane, then digested with trypsin and analyzed separately by LC-MS/MS. (TIF) [file pone.0032301.s003.tif]

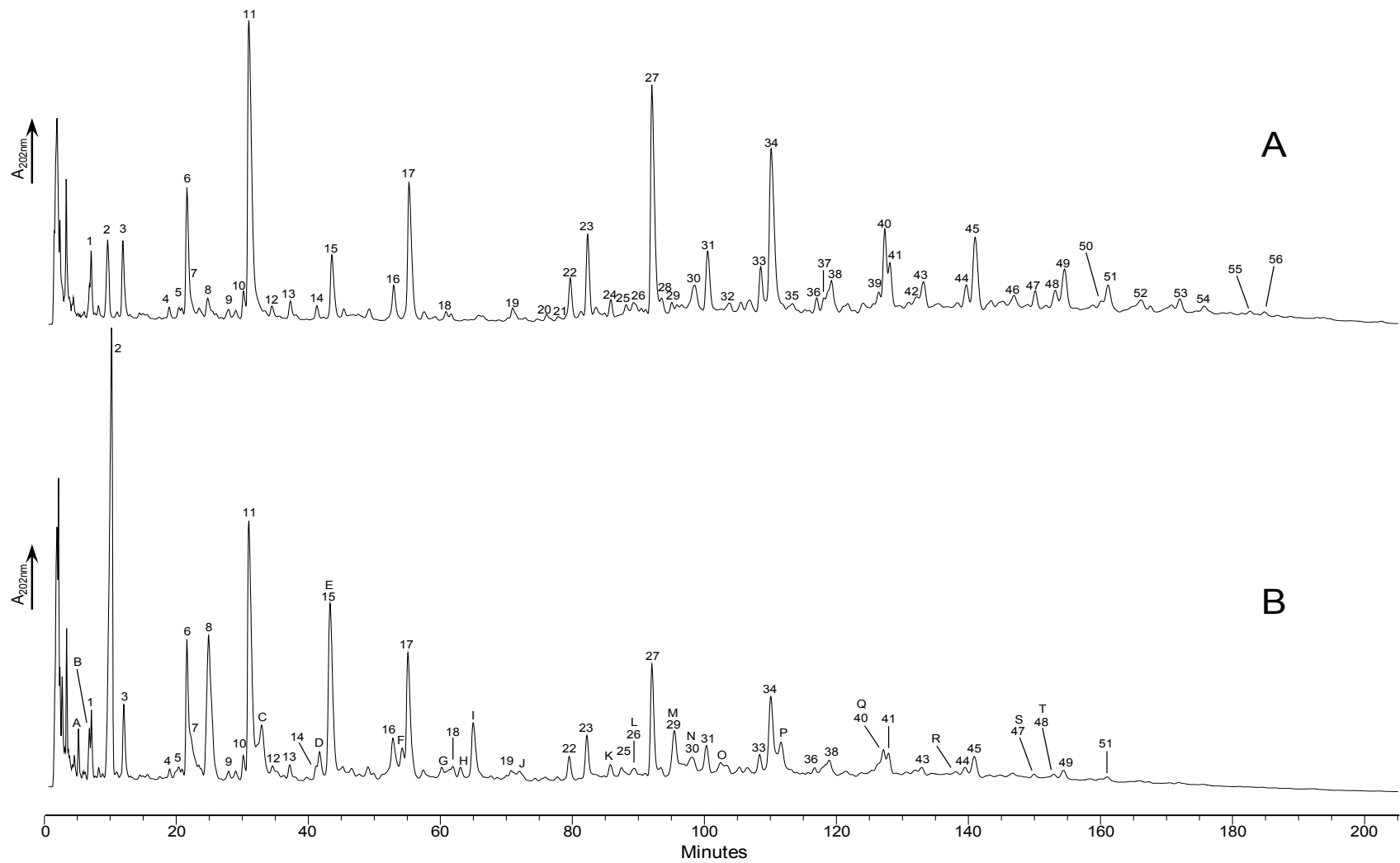

Figure S3

Supplement: Figure S3 — RP-HPLC profile of muropeptides obtained from L. casei BL23 PG digested by mutanolysin (A) or by mutanolysin and recombinant Lc-p75 (B). (PDF) [file pone.0032301.s004.pdf]

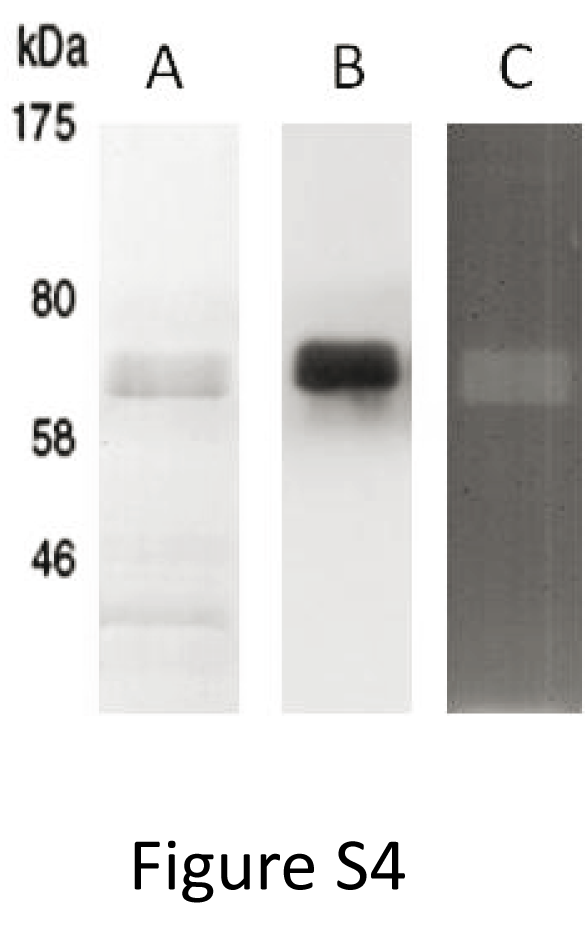

Supplement: Figure S4 — Detection of Strep-tagged Lc-p75 in a culture supernatant of the lcabl_02770 -negative mutant (PAR006). (A) SDS-PAGE gel, (B) Western Blot with monoclonal antibody directed against Strep-tag and (C) zymogram. (TIF) [file pone.0032301.s005.tif]
