# Supplementary material for: Analysis of the Peptidoglycan Hydrolase Complement of Lactobacillus casei and Characterization of the Major γ-D-Glutamyl-L-Lysyl-Endopeptidase
Source: PLoS One. 2012 Feb 27;7(2):e32301. doi: 10.1371/journal.pone.0032301 (PMC3288076; doi:10.1371/journal.pone.0032301)
Supplement: Table S1 — Bacterial strains and plasmids. (PDF) [file pone.0032301.s006.pdf]

Table S1. Bacterial strains and plasmids

Strain or plasmid	Characteristic(s)[a]	Source or reference
Lactobacillus casei		
BL23	Wild type strain	[1]
PAR 004	BL23 *lcabl_02770::lox72*	this work
PAR 005	BL23 containing pMSP::2770 plasmid	this work
PAR 006	BL23 *lcabl_02770::lox72* containing pMSP::2770StrepTag plasmid	this work
PAR 024	BL23 containing pMSP::2770StrepTag plasmid	this work
Escherichia coli		
TOP 10	*F- mcrA Δ(mrr-hsdRMS-mcrBC) φ80lacZΔM15 ΔlacX74 nupG recA1 araD139 Δ(ara-leu)7697 galE15 galK16 rpsL(Str^R) endA1 λ$^-$*	Invitrogen
TG1repA+	TG1 derivative with *repA* gene integrated into the chromosome, allowing replication of *Lactococcus lactis* plasmids	P. Renault, INRA, Jouy-en-Josas, France
PAR 003	TOP 10 strain containing pBad/His B::2770 plasmid	this work
**Plasmids**		
pNZ5319	Cm^r Em^r; pACYC184 derivative containing the *cat* gene under the control of the P_{32} constitutive promoter of *L. lactis* (*lox66*-P_{32}*cat*-*lox71* cassette)	[2]
pGhost Cre	Em^r; thermosensitive, Cre expression vector	[3]
pBad/His B	Amp^r, pBR322-derived expression vector carrying the *ara*BAD promoter (P_{BAD}) and allowing N-terminal 6xHis tag fusion	Invitrogen
pBad/His B::2770	pBad/His B derivative containing *lcabl_02770* gene without signal peptide sequence	this work
pMSP3545	Em^r ; shuttle vector carrying the *nisRK genes*, P_{nisA} promoter and start codon in *Nco*I site allowing translational fusion	[4]
pMSP::2770	pMSP3545 derivative containing *lcabl_02770*	this work
pMSP::2770StrepTag	pMSP3545 derivative containing *lcabl_02770* with a C-terminal Strep-TagII	this work

[a] Cm^r and Em^r indicate resistance to chloramphenicol and erythromycin, respectively.

References

1. Acedo-Felix E, Perez-Martinez G (2003) Significant differences between *Lactobacillus casei* subsp. *casei* ATCC 393T and a commonly used plasmid-cured derivative revealed by a polyphasic study. Int J Syst Evol Microbiol 53: 67-75.
2. Lambert JM, Bongers RS, Kleerebezem M (2007) Cre-lox-Based System for Multiple Gene Deletions and Selectable-Marker Removal in *Lactobacillus plantarum*. Appl Environ Microbiol 73: 1126-1135.
3. Fontaine L, Dandoy D, Boutry C, Delplace B, de Frahan MH, et al. (2010) Development of a versatile procedure based on natural transformation for marker-free targeted genetic modification in *Streptococcus thermophilus*. Appl Environ Microbiol 76: 7870-7877.
4. Bryan EM, Bae T, Kleerebezem M, Dunny GM (2000) Improved vectors for nisin-controlled expression in gram-positive bacteria. Plasmid 44: 183-190.
