# Supplementary material for: Analysis of the Peptidoglycan Hydrolase Complement of Lactobacillus casei and Characterization of the Major γ-D-Glutamyl-L-Lysyl-Endopeptidase
Source: PLoS One. 2012 Feb 27;7(2):e32301. doi: 10.1371/journal.pone.0032301 (PMC3288076; doi:10.1371/journal.pone.0032301)
Supplement: Table S2 — Primers used for cloning and validation. (PDF) [file pone.0032301.s007.pdf]

Table S2. Primers used for cloning and validation

Primer name	Sequence[a]	Plasmid
**Primers used for construction of deletion vector**		
2770-upF-DCO	5’-GTCACAGGTAATCAAGTTGG-3’	pNZ5319
2770-upR-DCO	5’-CTTTGCATCTACCATGTATG-3’	
2770-dwF-DCO	5’-CGTCCTTCACTATAAGGATAAC-3’	
2770-dwR-DCO	5’-ACACGATAAATGGAGGAAG-3’	
**Primers used for the validation and sequencing of deletion vector**		
SeqU1	5’-ATTATTCGTTTGATTTCG-3’	pNZ5319
SeqU2	5’-AACGCCTAAGACATTGTC-3’	
SeqD1	5’-TAGGAATCATTACCGAAG-3’	
SeqD2	5’-ATAGTTTACCCCGTCAGC-3’	
**Primers used for the construction of over-expression vectors**		
2770-pBAD-F	5’-CCGCTCGAGCTCAACGGGGACAGTCAATTACAAATCG-3’	pBad/His B
2770-pBAD-R	5’-CCCAAGCTTAGCTCTAGATTATAGTGAAGGACGAACAGC-3’	
2770-pMSP-F	5’-CATGCCATGGTAGATGCAAAGAAAGTATTG-3’	pMSP3545
2770-pMSPStrepTag-R	5’- GCTCTAGATTATTTTTCAAATTGTGGATGTGACCAA GCTGATAGTGAAGGACGAACAGC-3’	
**Primers used for the validation and sequencing of over-expression vectors**		
pBad-F	5’-ATGCCATAGCATTTTTATCC-3’	pBad/His B
pBad-R	5’-GATTTAATCTGTATCAGG-3’	
pMSP3545-F	5’-ACGGCTCTGATTAAATTCTG-3’	pMSP3545
pMSP3545-R	5’-CAATTGAACGTTTCAAGCC-3’	

[a] Restriction sites introduced in the primers are underlined
