# Supplementary material for: Analysis of the Peptidoglycan Hydrolase Complement of Lactobacillus casei and Characterization of the Major γ-D-Glutamyl-L-Lysyl-Endopeptidase
Source: PLoS One. 2012 Feb 27;7(2):e32301. doi: 10.1371/journal.pone.0032301 (PMC3288076; doi:10.1371/journal.pone.0032301)
Supplement: Table S3 — Structures, molecular masses and proportions of muropeptides obtained from L. casei BL23 PG digested by mutanolysin or by mutanolysin and recombinant Lc-p75. (PDF) [file pone.0032301.s008.pdf]

Table S3. Structures, molecular masses and proportions of muropeptides obtained from *L. casei* BL23 PG digested by mutanolysin or by mutanolysin and recombinant Lc-p75

Peak[a]	Proposed structure[b,]	Observed m/z	Calculated[c] $[M+Na]^+$	% of all peaks[d]	
				mutanolysin	mutanolysin and Lc-p75
1	Tri	848.22	848.39	1.69	1.06
2	Di	720.28	720.29	2.26	15.19
3	Tetra	919.41	919.42	2.18	1.95
4	Tri (Ac)	890.38	890.4	0.13	0.24
	Penta	991.3	991.44	0.13	
5	Tri (Ac)	890.35	890.4	0.50	0.36
6	Tri-N	962.43	962.43	4.14	3.92
7	Di (Ac)	762.36	762.3	0.44	1.87
8	Di (Ac)	762.31	762.3	0.79	7.59
9	Tetra (Ac)	961.37	961.43	0.35	0.27
10	Tetra (Ac)	961.49	961.43	0.75	0.63
11	Tetra-N	1033.44	1033.47	14.12	10.85
12	Tetra-D	1034.35	1034.45	0.47	0.25
13	Penta-D	1104.48	1104.5	0.59	0.44
14	Tri-N (Ac)	1004.51	1004.44	0.47	0.34
15	Tri-N (Ac)	1004.38	1004.44	2.66	2.36
16	Tetra-N (Ac)	1075.41	1075.48	1.53	2.13
17	Tetra-N (Ac)	1075.48	1075.48	6.13	5.58
18	Penta-D (Ac)	1146.58	1146.51	0.32	0.44
19	Tri-N-Tetra	1840.92	1840.85	0.62	0.32
20	Tri-D-Tetra (Ac) ou Tri-N-Tri-N	1883.77	1883.85	0.26	
21	Tri-N-Tetra-N missing GlcNAc	1751.83	1751.82	0.18	
22	Tetra-N-Tetra	1911.83	1911.89	1.62	0.88
23	Tri-N-Tetra-N	1954.86	1954.89	3.01	1.62
24	Tetra-D-Tetra (Ac)	1954.78	1954.88	0.58	
25	Tetra-N-Tetra-N missing GlcNac	1822.85	1822.85	0.46	0.34
26	Tri-N-Tetra (Ac)	1882.82	1882.86	0.76	0.19
27	Tetra-N-Tetra-N	2025.93	2025.93	9.73	4.26
28	Tri-D-Tetra (2-Ac) ou Tri-N-Tri-N (Ac)	1925.77	1925.86	0.62	
29	Tetra-N-tetra (Ac)	1953.87	1953.9	0.11	0.39
	Tetra-D-Tetra-N	2026.87	2026.92	0.11	
	Penta-D-Tetra-N	2096.93	2096.97	0.11	
30	Tetra-N-tetra (Ac)	1953.95	1953.9	1.87	1.03
31	Tri-N-Tetra-N (Ac)	1996.68	1996.91	2.70	1.38
32	Tri-N-Tetra-N (Ac)	1996.75	1996.91	0.46	
33	Tetra-N-Tetra-N (Ac)	2067.9	2067.94	1.87	0.71
34	Tetra-N-Tetra-N (Ac)	2067.94	2067.94	9.18	4.16
35	Penta-N-Tetra-N (Ac)	2138.97	2138.98	0.56	
36	Tetra-N-tetra (2-Ac)	1995.95	1995.91	0.54	0.27
37	Tetra-N-Tetra-N-Tetra	2903.99	2904.35	0.51	
38	Tri-N-Tetra-N (2-Ac)	2038.82	2038.92	1.03	1.28
	Tri-N-Tetra-N-Tetra-N	2947.14	2947.36	1.03	
39	Tetra-N-Tetra-N (2-Ac)	2109.79	2109.95	0.37	
	Tetra-N-Tetra-N-Tetra-N missing GlcNac	2815.08	2815.31	0.37	
40	Tetra-N-Tetra-N-Tetra-N	3018.23	3018.39	3.33	0.89
41	Tetra-N-Tetra-N (2-Ac)	2110.17	2109.95	1.95	0.82

42	Tetra-N-Tetra-N-Tetra (Ac)	2946.28	2946.36	0.30	
	Tetra-N Anhydro	1013.49	1013.44	0.30	
43	Tri-N-Tetra-N-Tetra-N (Ac)	2989.43	2989.37	1.32	0.37
44	Tetra-N-Tetra-N-Tetra-N (Ac)	3060.38	3060.4	1.10	0.43
45	Tetra-N-Tetra-N-Tetra-N (Ac)	3060.42	3060.4	3.83	1.11
46	Tri-N-Tetra-N-Tetra-N (2-Ac)	3031.27	3031.38	0.92	
47	Tetra-N-Tetra-N-Tetra-N-Tetra-N	4010.44	4010.86	0.77	0.04
48	Tetra-N-Tetra-N-Tetra-N (2-Ac)	3102.6	3102.41	0.96	0.11
49	Tetra-N-Tetra-N-Tetra-N (2-Ac)	3102.46	3102.41	2.31	0.47
50	Tri-N-Tetra-N-Tetra-N (3-Ac)	3073.26	3073.39	0.48	
51	Tetra-N-Tetra-N-Tetra-N-Tetra-N (Ac)	4052.84	4052.87	1.65	0.22
52	Tetra-N-Tetra-N-Tetra-N (3-Ac)	3144.38	3144.42	0.45	
	Tri-N-Tetra-N-Tetra-N-Tetra-N (2-Ac)	4023.91	4023.85	0.45	
53	Tetra-N-Tetra-N-Tetra-N-Tetra-N (2-Ac)	4094.78	4094.88	0.73	
54	Tetra-N-Tetra-N-Tetra-N-Tetra-N-Tetra-N (Ac)	5045.2	5045.34	0.48	
55	Tetra-N-Tetra-N-Tetra-N-Tetra-N (3-Ac)	4136.79	4136.89	0.20	
56	Tetra-N-Tetra-N-Tetra-N-Tetra-N-Tetra-N (2-Ac)	5087.39	5087.35	0.20	
**New forms of partially digested muropeptides by Lc-p75**					
A	Di missing Ac	678.15	678.28		0.64
B	Di missing GlcNac	517.16	517.21		0.89
C	Tri-N-(A-K)-N	1275.53	1275.6		3.34
D	Tetra-N-(A-K)-N	1346.56	1346.65		1.04
E	Tetra-N-(A-K)-N	1346.59	1346.65		6.06
F	Tri-N-(A-K)-N (Ac)	1317.37	1317.62		1.27
G	Tetra-D-(A-K)-N	1347.43	1347.63		0.41
H	Tetra-N-(A-K)-N (Ac)	1388.73	1388.65		0.37
I	Tetra-N-(A-K)-N (Ac)	1388.84	1388.65		2.62
J	Tetra-N-(A-K)-N-(A-K)-N (Ac)	1701.92	1701.82		0.52
K	Tri-N-Tetra-N-(A-K)-N	2268.08	2268.06		0.46
L	Tri-N-Tetra-N-(A-K)-N-(A-K)-N	2581.18	2581.24		0.19
M	Tetra-N-Tetra-N-(A-K)-N	2339.12	2339.11		1.98
N	Tetra-N-Tetra-N-(A-K)-N-(A-K)-N	2652.05	2652.28		0.41
O	Tri-N-Tetra-N-(A-K)-N (Ac)	2310.18	2310.07		0.32
	Tri-N-Tetra-N-(A-K)-N-(A-K)-N (Ac)	2623.35	2623.25		0.32
P	Tetra-N-Tetra-N-(A-K)-N (Ac)	2381.08	2381.12		1.79
Q	Tetra-N-Tetra-N-(A-K)-N (2-Ac)	2423.01	2423.13		0.28
R	Tetra-N-Tetra-N-Tetra-N-(A-K)-N (Ac)	3373.3	3373.58		0.08
	Tetra-N-Tetra-N-Tetra-N-(A-K)-N-(A-K)-N (Ac)	3686.45	3686.75		0.08
S	Tetra-N-Tetra-N-Tetra-N-(A-K)-N (2-Ac)	3415.62	3415.59		0.04
	Tetra-N-Tetra-N-Tetra-N-(A-K)-N-(A-K)-N (2-Ac)	3728.7	3728.76		0.04
T	Tetra-N-Tetra-N-Tetra-N-(A-K)-N (2-Ac)	3415.53	3415.59		0.11

[a]Peak numbers refer to Figure S2.

[b]Di, disaccharide dipeptide (L-Ala-D-iGln); Tri, disaccharide tripeptide (L-Ala-D-iGln-L-Lys); Tetra, disaccharide tetrapeptide (L-Ala-D-iGln-L-Lys-D-Ala); Penta, disaccharide pentapeptide (L-Ala-D-iGln-L-Lys-D-Ala-D-Lac); Disaccharide, GlcNAc-MurNAc; Ac, acetylation on MurNAc, iGln, isoglutamine; N, D-Asn; A, D-Ala; K, L-Lys.

[c]Sodiated molecular ions were the most abundant ones on MALDI-TOF mass spectra for all muropeptides.

[d]Percentage of each peak was calculated as the ratio of the peak area over the sum of areas of all the peaks identified in the corresponding chromatogram.
