# Supplementary material for: Analysis of the Peptidoglycan Hydrolase Complement of Lactobacillus casei and Characterization of the Major γ-D-Glutamyl-L-Lysyl-Endopeptidase
Source: PLoS One. 2012 Feb 27;7(2):e32301. doi: 10.1371/journal.pone.0032301 (PMC3288076; doi:10.1371/journal.pone.0032301)
Supplement: Table S4 — Primers used for RT-PCR experiments. (PDF) [file pone.0032301.s009.pdf]

Table S4 : Primers used for RT-PCR experiments.

Primer name	Locus tag	Sequence
LcTuf-F	LCABL_15580 (tuf)	5'- TTCCTGTTATCCGTGGTTCTG -3'
LcTuf -R	LCABL_15580 (tuf)	5'- AATCACACCGGTAACATCAGTG -3'
LcLys-F	LCABL_11280	5'- CCACACTAGTGATCCCGAAAC -3'
LcLys-R	LCABL_11280	5'- TCTGGACTCCATCAATCACTTC -3'
LcAcmA-F	LCABL_12760	5'- CGTTAAAAATAATTCCGCTTGC -3'
LcAcmA-R	LCABL_12760	5'- AGTAGCATAGCCATCCGTCTTC -3'
LcAmi-F	LCABL_17510	5'- GGTTAATCAACCGGTTAACGTC -3'
LcAmi-R	LCABL_17510	5'- TTTTTCATAGTCGGTGCCATC -3'
Lc230-F	LCABL_00230	5'- TTGGTTGCTGCAGTTACCTTAG -3'
Lc230-R	LCABL_00230	5'- GATTTCTGGGTTTCCAATGAAG -3'
Lc2770-F	LCABL_02770	5'- GATGTTGCTGTCTCCAGTGC -3'
Lc2770-R	LCABL_02770	5'- GCCGTTCAAAAAGGCATAATAC -3'
LcTrsG-F	LCABL_05960	5'- ACTTAGCAAACGTTGGTTGTTG -3'
LcTrsG-R	LCABL_05960	5'- CTGGCATCTGCTCCAAATTC -3'
LcLcP45-F	LCABL_21960	5'- TATGAGCCAAGCGTACTTTGAG -3'
LcLcP45-R	LCABL_21960	5'- CACTTGTTGCGACAAAGAGC -3'
LcAtlh-F	LCABL_04610	5'- TTACGCGCATTATCACAACAG -3'
LcAtlh-R	LCABL_04610	5'- ATCACACTCAAAATCGTCTTGC -3'
LcPs356-F	LCABL_13470	5'- GCGAAGTATCAGGGAGCAAG -3'
LcPs356-R	LCABL_13470	5'- TGCCAAATAGCTACCCCATC -3'
LcIslA-F	LCABL_02350	5'- TTTGCGAACACTTTGGACAG -3'
LcIslA-R	LCABL_02350	5'- TCGTCGTTGCTTGTTGAATC -3'
LcLysozyme-F	LCABL_12360	5'- CAGTCGGGTCATTTATGCTG -3'
LcLysozyme-R	LCABL_12360	5'- TACCCATCAGCAACACTTGG -3'
LcLys2-F	LCABL_10020	5'- GCACGTTGGTATCATTCACG -3'
LcLys2-R	LCABL_10020	5'- TGTGTGCGGTAATAGGCTTG -3'
